# Gastric and Duodenal Antiulcer Activity of Alkaloids: A Review

**DOI:** 10.3390/molecules13123198

**Published:** 2008-12-17

**Authors:** Heloina de Sousa Falcão, Jacqueline Alves Leite, José Maria Barbosa-Filho, Petrônio Filgueiras de Athayde-Filho, Maria Célia de Oliveira Chaves, Marcelo Dantas Moura, Anderson Luiz Ferreira, Ana Beatriz Albino de Almeida, Alba Regina Monteiro Souza-Brito, Margareth de Fátima Formiga Melo Diniz, Leônia Maria Batista

**Affiliations:** 1 Laboratório de Tecnologia Farmacêutica, Universidade Federal da Paraíba - UFPB, Cx. Postal 5009, 58051-970, João Pessoa, PB, Brazil; E-mail: heloinafalcao@yahoo.com.br (H. S-F.), jacq_pet@hotmail.com (J-A. L.), jbarbosa@ltf.ufpb.br (J-M. B-F.), athayde_filho@pesquisador.cnpq.br(F. A-F.), cchaves@ltf.ufpb.br (M-C. O-C.), tetelodantas@bol.com.br (M-D. M.), margareth@ccs.ufpb.br (M. F-F.), leoniab@uol.com.br (L-M. B.); 2 Laboratório de Produtos Naturais, Universidade Estadual de Campinas - UNICAMP, Cx. Postal 6109, 13083-970, Campinas, SP, Brazil; E-mails: domcarecone@yahoo.com.br (A-L. F.), anabia5@yahoo.com.br (A-A. A.), abrito@unicamp.br (A-M. S-B.)

**Keywords:** Alkaloids, Antiulcer activity, Peptic ulcer, Review.

## Abstract

Peptic ulcer disease is a deep gastrointestinal erosion disorder that involves the entire mucosal thickness and can even penetrate the muscular mucosa. Numerous natural products have been evaluated as therapeutics for the treatment of a variety of diseases, including this one. These products usually derive from plant and animal sources that contain active constituents such as alkaloids, flavonoids, terpenoids, tannins and others. The alkaloids are natural nitrogen-containing secondary metabolites mostly derived from amino acids and found in about 20% of plants. There has been considerable pharmacological research into the antiulcer activity of these compounds. In this work we review the literature on alkaloids with antiulcer activity, which covers about sixty-one alkaloids, fifty-five of which have activity against this disease when induced in animals.

## Introduction

For over a century, peptic ulcer disease has been one of the leading causes of gastrointestinal surgery, with high morbidity and mortality rates. The prevalence of gastrointestinal ulcers differs around the world: duodenal ulcers are dominant the Western populations and gastric ulcers are more frequent in Asia, especially in Japan. As the prevalence of this disease increases over time, one would expect peptic ulcers to continue to have a significant global impact in the basic health and economic systems and in patients’ life quality [1].

Peptic ulcers are a deep gastrointestinal erosion disorder that involves the entire mucosal thickness, penetrating the muscular mucosa [2]. For decades it was believed that gastrointestinal ulcerations were caused by the excessive secretion of gastric acid, but many patients presenting such ulcerations had normal acid secretion rates [3]. Then, researchers reported that peptic ulcers were been caused by an imbalance between the aggressive factors and a number of known defense mechanisms. Exogenous aggressive factors such as smoke, anti-inflammatory drugs, alcohol, stress, fatty foods and *Helicobacter pylori* infections triggered tissue necrosis through mucosal ischemia, free radical generation and cessation of nutrient delivery, hydrochloric acid together with pepsin, pancreatic enzymes and bile decreased the defense mechanisms of gastrointestinal mucosa such as the intercellular junctions, local blood flow, mucus/bicarbonate secretion and cellular growth [2,4,5].

In recent years, a large advance in chemical and pharmacological studies has contributed to the knowledge about new therapeutically active compounds obtained from the natural products [6]. These compounds can be used directly as leads for the development of new medicines or as pharmacological tools to discover new active compounds, so they can be life-saving or determine the quality of life in long-lasting diseases [7,8]. However, the incorrect use of the natural products offers dangers to society, so it is important to identify the active compounds, linking its structure with the biological activity and report the correct manner to use them with regards to dose, route of administration and frequency of use [9].

The natural active compounds classes or secondary metabolites as alkaloids, flavonoids, terpenoids, tannins and others have attracted researchers to investigate their chemical, toxicological and pharmacological features. The alkaloids represent a group of natural products that has had a major impact throughout history on the economic, medical, political and social affairs of humans. They are a diverse group of low molecular weight nitrogen-containing compounds derived mostly from amino acids [10]. These secondary metabolites are found in about 20 % of plant species and they classified as true alkaloids, which have nitrogen atoms in heterocyclic rings, protoalkaloids, which do not have the nitrogen atom(s) in heterocyclic rings and pseudoalkaloids, which don’t derive from amino acids but may have nitrogen atoms in heterocyclic rings [11].

Several alkaloids are being used in therapeutics and as pharmacological tools. A wide range of biological activities of alkaloids have been reported: emetic, anti-cholinergic, antitumor, diuretic, sympathomimetic, antiviral, antihypertensive, hypnoanalgesic, antidepressant, miorelaxant, antitussigen, antimicrobial and anti-inflammatory [11]. However, the alkaloids and other natural compounds have complex activities and it is necessary to analyze pharmacological activities in the general tissues, linking the structure with the activity presented. It is common to find pharmacological results where a single experimental model generalizes a biological answer, but these can’t be accepted because all the pathologies in question are also complex and it is necessary to investigate specific experimental models. 

In the course of our continuing search for bioactive natural products from plants, we have recently published reviews on crude plant extracts and plant-derived compounds with the following potential activities and uses: as inhibitors of mammary, uterine cervical and ovarian neoplasia [12,13,14]; as inhibitors of HMG CoA reductase [15]; with central analgesic activity [16]; employed in prevention of osteoporosis [17]; for the treatment of Parkinson’s disease [18]; antileishmanial, hypoglycemic and anti-inflammatory activities [19,20,21,22]; as inhibitors of the acetylcholinesterase enzyme [23], inhibitors of the angiotensin-converting enzymes [24], with giardicidal and antileprotic activities [25,26] and plant extracts with antiulcer activity [27].

In this article, we have reviewed some reports about alkaloids with antiulcer activity in the specialized literature pubished up to December 2007. The search was carried out on data banks such as SciFinder Scholar, Periódicos CAPES, Pubmed and NAPRALERT (acronym for Natural Products ALERT - University of Illinois in Chicago, U.S.A.). The references were consulted for details of the experimental models used for testing the alkaloids against peptic ulcer, activities, route of administration, organism tested and subtypes of alkaloids studied.

## Alkaloids studied in models that investigate the antiulcer activity

For this review we identified sixty-one alkaloids studied in models that evaluate the antiulcer activity and distributed among thirteen subtypes: imidazole, indole, isoquinoline, non-nitrogen heterocycle alkaloid, phenylalkylamide, piperidine, pyrazine, pyridine, pyrrolidine, pyrrolizidine, quinolizidine, steroid and tropane alkaloids. Fifty-five of these alkaloids have reported antiulcer activity (see Table 1).

Among the different alkaloids showing potent pharmacological properties are the narcotic analgesic morphine, the antimicrobial berberine and the sympathomimetic ephedrine. These isoquinoline alkaloids occur mainly in the Papaveraceae, Berberidaceae and Ephedraceae families [10]. The compounds morphine and ephedrine also have confirmed antiulcer activity, inhibiting gastric lesions induced by reserpine, aspirin or indomethacin [28,29]. Berberine didn’t display this activity towards ethanol induced ulcers [30,31,32], but 7,8-dihydro-8-hydroxypalmatine, a new type of protoberberine alkaloid obtained from the bark of *Enantia chlorantha*, accelerated ulcer-healing and increased the gastric mucus production after the lesions have been caused by acetic acid, HCl/ethanol or absolute ethanol and it also has been investigated in the pylorus ligature model [33,34]. Other isoquinoline alkaloids isolated from *Coptidis* rhizome, coptisine and 8-oxocoptisine, showed protection of gastric mucosa similar to that offered by gastroprotective medicines such as cimetidine and sucralfate [30,35,36].

Tropane alkaloids are dicyclic compounds formed by condensation of a pyrrolidine precursor amino acid (ornithine) with three acetate-derived carbon atoms. Some of these alkaloids such as atropine and scopolamine constitute an important class of anticholinergic compounds derived from plants that occur in several *Atropa* and *Datura* species [10]. Clinically, they are used to block the muscarinic activity of acetylcholine showing antispasmodic and antisecretory effects in the treatment of spastic colitis, gastroenteritis and peptic ulcer. They also are useful pharmacological tools to discover new active principles with gastrointestinal tract actions [37,38,39,40]. Anisodamine and anisodine are analogs of atropine and they were evaluated as protectors of gastric mucosa against the damaging effects induced in rats by indomethacin, reserpine, stress, pylorus ligature, acetic acid or absolute ethanol. In this research, these compounds inhibited the lesions caused by aggressor agents and altered the gastric acid secretion through increase of luminal gastric output of basal bicarbonate and pH [39,41,42,43]. Another well known tropane alkaloid, cocaine, showed antiulcer activity against ulcers induced by reserpine in rats when it was used in the dose of 10 mg/kg by oral route of administration [29]. This substance is obtained of the *Erythroxylum coca* leaves and has multiple actions in the central and peripheral nervous system. It is a psychomotor stimulant with a strong abuse potential and has ability to dominate or decreasing behaviors such as eating and sleeping.

A pyridine alkaloid well known in society is nicotine, which is found in the Solanaceae family, mainly in the dried leaves of the tobacco plant *Nicotiana tabacum* Linné. This substance acts on the nicotinic receptors of acetylcholine in autonomic ganglia, adrenal medulla, neuromuscular junction and brain of mammals. The chronic use of nicotine may result in psychologic and physical dependence. However, this alkaloid protected the stomach from damage induced by aspirin by decreasing hemorrhages and increasing the pH gradient/gastric fluid volume [44].

Quinolizidine alkaloids such as matrine, 13-alpha-hydroxymatrine and oxy-matrine were isolated from *Sophora flavescens* (Fabaceae) and they were experimentally tested for inhibition of gastric ulcers induced by pylorus ligature, water immersion stress and indomethacin. These alkaloids decreased the acid secretion and inhibited the gastric motility [45,46,47,48,49].

A piperidine alkaloid is piperine, which has a pungent taste and was studied in connection with the gastric mucosa damage caused by stress, indomethacin, ethanol or pylorus ligature in rats or mice. This substance protected the stomach against ulceration by decreasing the volume of gastric juice, gastric acidity and pepsin-A activity in doses of 1.5 mg/kg and 25 mg/kg after intravenous and oral administrations, respectively [50,51]. Capsaicin is a phenylakylamide alkaloid and it also has a pungent taste. This substance provides a selective probe for mechanisms involving slowly conducting primary afferent neurons. In the stomach, capsaicin (0.3 nanolmol/kg - 10 mg/kg) stimulates these neurons and signalizes for protection inhibiting the acid secretion, stimulating the alkali/mucus secretions and mainly increasing the gastric mucosal blood flow which help in prevention and healing of ulcers against injury caused by aggressive agents. However, neurotoxic doses of capsaicin have augmented the susceptibility of gastric mucosa to injury caused by strong irritants [52,53].

Steroidal alkaloids have been found in the Apocynaceae, Buxaceae, Liliaceae and Solanaceae families. In this review, pachystermine A, pachysamine A, epipachysamine A, pachysandrine A and spiropachysine obtained from *Pachysandra terminalis* (Buxaceae), used in Ainu folk medicine for gastrointestinal diseases, were investigated for their preventive effects on gastric lesions induced by water-immersion stress in mice and the results this evaluation suggested that these alkaloids may contribute partly to the traditional use of the plants for the gastrointestinal complaints [54].

An important class of alkaloid is the indole constituted by melatonin, cantinone, hirsuteine, reserpine, lysergic acid (LSD), yohimbine and nigakinone. Melatonin has been found in algae and humans. This human hormone is secreted by pineal gland and gastrointestinal cells. Moreover, it has antiulcer activity because showed to protect the gastric mucosa from the damage caused by ischemia-reperfusion and absolute ethanol through of the attenuation of the gastric blood flow failed and scavenging of free radicals [55]. Reserpine was isolated of *Rauvolfia serpentina* (Apocynaceae) and didn’t show antiulcer effect in the dose of 2.5 mg/kg when the gastric ulcers were induced by stress in mice [46], while yohimbine, obtained of *Pausinystalia yohimbe* (Rubiaceae), was active in the reduction of gastrointestinal ulceration [56]. Other alkaloids such as hirsutine, hirsuteine, and rhynchophylline isolated from the domestic plant *Uncaria rhynchophylla* Miq. showed mild central depressive, anti-spasmodic and hypotensive effects in mice or rats. These substances also were effective in the dose of 60 mg/kg against gastric lesions, while isorhynchophylline didn´t have a preventive effect on the development of gastric erosions in mice [56]. Nigakinone and methylnigakinone are also indole alkaloids and they can be found in *Picrasma quassioides*, *P. ailanthoides* or *Ailanthus altissima*. These substances had antiulcer effects associated with decreases in gastric acid/pepsin secretions and protection of the mucous membrane [57,58]. Cantin-6-one and 4-methoxycantinone are alkaloids extracted from Simaroubaceae plants. These substances were found in *Quassia amara, Simaba multiflora, S. polyphylla, S. feruginea* and *Eurycoma longifolia* which are popularly indicated for gastrointestinal disorders, obesity, anti-inflammatory, stimulant of the intestinal motility and central nervous system activities. These compounds were effective against gastric lesions induced by ethanol and indomethacin [59].

The pyrrolizidine alkaloids integerrimine, retrorsine, senecionine, usaramine and seneciphylline were extracted from *Senecio brasiliensis*. These alkaloids demonstrate significant activity in acute and chronic gastric ulcers in the dose of 12.5 mg/kg. These alkaloids increased free mucus and prostaglandin in the mucosal gastric. Moreover, they showed a reduction of exfoliation of superficial cells, hemorrhages and blood cell infiltration that can be mediated by increase in gastrin secretion and mRNA expression of epidermal growth factors [60].

**Table 1:** Alkaloids with gastrointestinal antiulcer activity

Substance	Experimental models/dose – Route of administration	Organism tested	Effect
**Imidazole alkaloids**			
Allantoin	Phenylbutazone induced ulcer/6 g/kg – gastric intubation	Rat	Active [61]
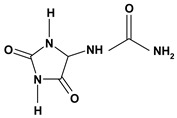	*/oral	Human adult	* [62]
Histamine, *para*-coumaroyl	*/ 100 mg/kg – intragastric	Mouse	Equivocal [63]
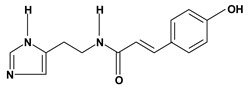	50% ethanol induced gastric ulcer/2.5; 10 and 20 mg/kg – oral and intraperitoneal	Mouse	Active [59]
4-Methoxycantin-6-one 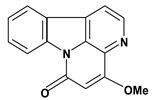	50% ethanol induced gastric ulcer/ 10 and 20 mg/kg – oral and intraperitoneal	Mouse	Active [59]
Hirsuteine 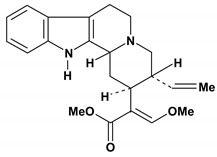	*/60 mg/kg – intraperitoneal	Mouse	Active [56]
Hirsutine 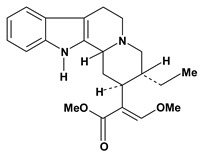	*/60 mg/kg – intraperitoneal	Mouse	Active [56]
LSD-25 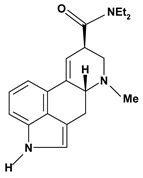	5-HT induced gastric ulcers/30mg/kg – intramuscular	Guinea pig	Active [64]
Melatonin	*/intragastric	Rat	Active [55]
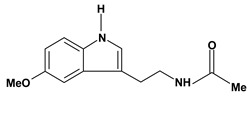	Ischemia-reperfusion induced ulcer/intragastric	Rat	Active [55]
Nigakinone	*/*	Rat	Active [57]
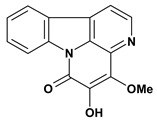	Pylorus-ligated induced ulcer/31.3 mg/kg - *	Rat	Active [57]
Nigakinone, methyl 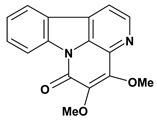	Pylorus-ligated induced ulcer/62.5 mg/kg - *	Rat	Active [58]
Reserpine	Stress induced ulcer/2.5 mg/kg –subcutaneous	Mouse	Inactive [46]
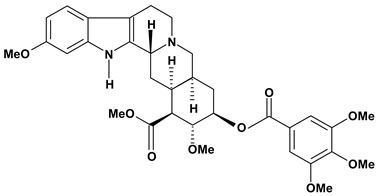			
Rhynchophylline 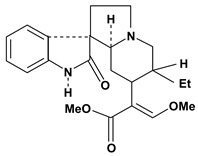	*/60 mg/kg – intraperitoneal	Mouse	Active [56]
Rhynchophylline, iso 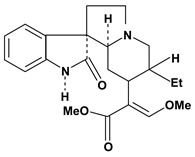	*/60 mg/kg – intraperitoneal	Mouse	Inactive [56]
Tabersonine 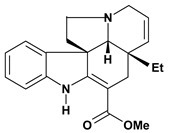	*/50 mg/kg – intragastric	Rat	Active [34]
Vinpocetine 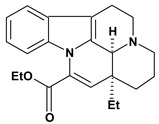	*/10 mg/kg – oral	Human adult	Active [65]
Yohimbine, beta 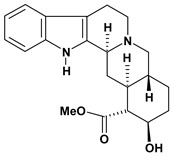	*/20 mg/kg – intraperitoneal	Mouse	Active [56]
**Isoquinoline alkaloids**			
Berberine	Ethanol induced gastric ulcer/25 mg/kg – intragastric	Rat	Inactive [30]
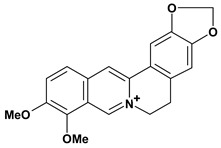	*/15 mg/kg – gastric intubation	Rat	Weak activity [31]
	Pylorus-ligated induced ulcer/10 mg/kg – oral	Rat	Inactive [32]
Berberine, oxy 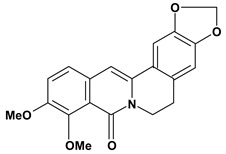	Ethanol induced gastric ulcer/50 mg/day - intragastric	Rat	Inactive [30]
Cathinone 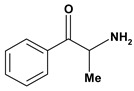	*/intragastric	Rat	Active [66]
Cathinone, (-)	Aspirin induced gasric ulcer/10 mg/kg - intragastric	Rat	Active [28]
	Indomethacin induced gastric ulcer/10 mg/kg - intragastric	Rat	Active [28]
	Phenylbutazone induced gastric ulcer/10 mg/kg - intragastric	Rat	Active [28]
Cathinone, (+)	Aspirin induced gasric ulcer/10 mg/kg - intragastric	Rat	Active [28]
	Indomethacin induced gastric ulcer/10 mg/kg - intragastric	Rat	Active [28]
	Phenylbutazone induced gastric ulcer/10 mg/kg - intragastric	Rat	Active [28]
Coptisine	Ethanol induced gastric ulcer/0.1 mg/kg - intragastric	Rat	Active [30]
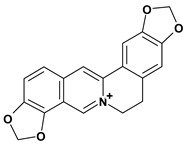	80% ethanol induced ulcer/0.1 mg/kg - intragastric	Rat	Active [35]
	80% ethanol induced ulcer/0.1 mg/kg - intragastric	Rat	Active [36]
Coptisine, 8-oxo	Ethanol induced gastric ulcer/0.1 mg/kg - intragastric	Rat	Active [30]
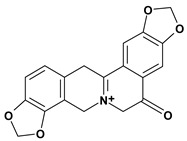	80% ethanol induced ulcers/0.1 mg/kg - intragastric	Rat	Active [35]
Corydaline 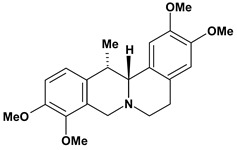	*/15 mg/kg - oral	Human adult	Active [67]
Corydaline, dehydro	*/40 mg/kg - oral	Mouse	Active [68]
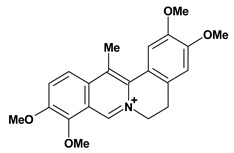	*/*	Bullfrog	Active [68]
	Pylorus-ligated induced gastric ulcer/oral	Rat	Active [69]
	Stress induced gastric ulcer/oral	Rat	Active [69]
	Pylorus-ligated induced gastric ulcer/subcutaneous	Rat	Active [69]
	Stress induced gastric ulcer/subcutaneous	Rat	Active [69]
	Histamine induced ulcers of duodenum/oral	Guinea pig	Active [69]
	Histamine induced ulcers of duodenum/subcutaneous	Guinea pig	Inactive [69]
	Reserpine induced gastric ulcer/oral	Rat	Active [69]
	Reserpine induced gastric ulcer/subcutaneous	Rat	Inactive [69]
Corydamine 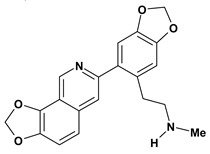	*/*	*	Active [70]
Corydamine, tetrahydro 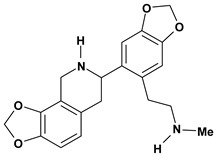	*/*	*	Active [71]
Ephedrine, nor- *n*-formyl (-)	Aspirin induced ulcers/5 mg/kg - intragastric	Rat	Active [28]
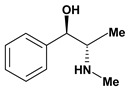	Indomethacin induced ulcers/10 mg/kg - intragastric	Rat	Active [28]
Ephedrine, nor- *n*-formyl (+) 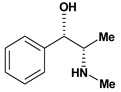	Aspirin induced ulcers/5 mg/kg - intragastric	Rat	Active [28]
Glaziovine	Histamine induced ulcers/5 mg/kg - intraperitoneal	Guinea pig	Active [72]
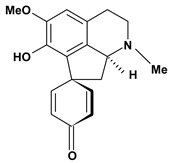	Reserpine induced ulcers/5 mg/kg - intraperitoneal	Rat	Active [72]
	Serotonin induced ulcers/5 mg/kg - intraperitoneal	Rat	Active [72]
	Restraint stress induced ulcers/5 mg/kg - intraperitoneal	Rat	Active [72]
	Pylorus-ligated induced ulcers/5 mg/kg - intraperitoneal	Rat	Active [72]
	*/10 mg/person - oral	Human adult	Active [73]
Morphine 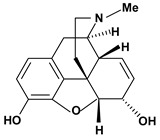	Reserpine induced ulcers/10 mg/kg - oral	Rat	Active [29]
Palmatine, 7-8-dihydro: 8-hydroxy	Ethanol induced gastric ulcers/50 mg/kg - intragastric	Rat	Active [33]
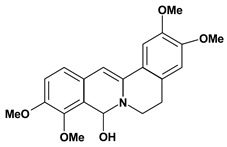	Pylorus-ligated induced ulcers/50 mg/kg - intragastric	Rat	Active [33]
	Acetic acid induced ulcers/80 mg/kg - intragastric	Rat	Active [33]
	*/100 mg/kg - intragastric	Rat	Active [34]
**Non- nitrogen heterocycle alkaloid**			
Chlorophyll	Pylorus-ligated induced ulcers/1 g/kg - intravenous	Rat	Active [74]
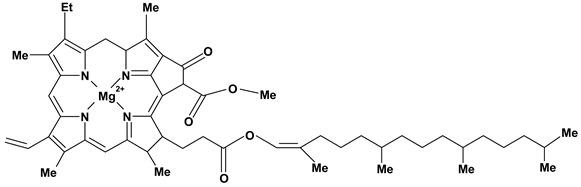			
**Phenylalkylamide alkaloid**			
Capsaicin	Ethanol induced gastric ulcer/0.3 nanomol/kg - intragastric	Rat	Active [52]
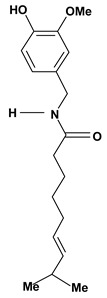	80% ethanol induced ulcer/2 mg/kg - intragastric	Rat	Active [75]
	80% ethanol induced ulcer/2 mg/kg - subcutaneous	Rat	Active [75]
	Pylorus-ligated induced ulcer/1 μg/kg - intragastric	Rat	Active [76]
	Aspirin induced ulcer/0.6 μg/kg - intragastric	Rat	Active [77]
	Ethanol induced ulcer/0.105 μg/kg - intragastric	Rat	Active [77]
	HCl induced ulcer/0.1 μg/kg - intragastric	Rat	Active [77]
	Acetic acid induced ulcer/10 mg/kg - intragastric	Rat	Active [78]
	*/0.5 mg/kg - intragastric	Rat	Active [53]
**Piperidine alkaloid**			
Piperine	Aspirin induced ulcer/1,5 mg/kg - intravenous	Rabbit	Active [50]
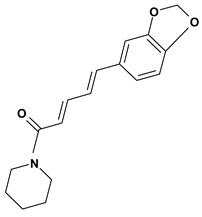	Restraint stress induced ulcer/25 mg/kg - intragastric	Rat	Active [51]
	Indomethacin induced ulcer/25 mg/kg - intragastric	Rat	Active [51]
	HCl/ethanol induced gastric ulcer/25 mg/kg - intragastric	Rat	Active [51]
	Pylorus-ligated induced ulcer/25 mg/kg - intragastric	Rat	Active [51]
	Restraint stress induced ulcer/25 mg/kg - intragastric	Mouse	Active [51]
	Indomethacin induced ulcer/25 mg/kg - intragastric	Mouse	Active [51]
	HCl/ethanol induced gastric ulcer/25 mg/kg - intragastric	Mouse	Active [51]
	Pylorus-ligated induced ulcer/25 mg/kg – intragastric	Mouse	Active [51]
**Pyrazine alkaloid**			
Ligustrazine	Water-immersion stress induced ulcer/intragastric	Rat	Active [79]
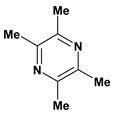	Restraint stress induced ulcer/intragastric	Rat	Active [79]
**Pyridine alkaloids**			
Gentianine	Pylorus-ligated induced ulcers/100 mg/kg - oral	Rat	Weak activity [80]
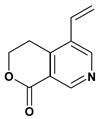	Water-immersion stress-induced ulcers/100 mg/kg - oral	Rat	Active [80]
Mallorepine 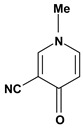	Stress induced gastric ulcer/300 mg/kg - subcutaneous	Mouse	Inactive [81]
Nicotine 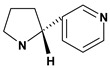	Aspirin induced ulcer/1mg/animal - intragastric	Rat	Active [44]
Pyrrolidine alkaloid			
Cuscohygrine 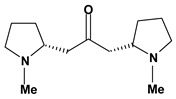	*/*	Rat	Active [82]
**Pyrrolizidine alkaloids**			
Interregimine	Hypothermic restraint stress induced gastric ulcer/12.5 mg/kg of crude alkaloid extract - oral	Mouse	Active [60]
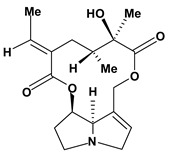	Ethanol induced gastric ulcer/12.5 mg/kg of crude alkaloid extract - oral	Rat	Active [60]
	Cysteamine induced duodenal ulcer/12.5 mg/kg of crude alkaloid extract - oral	Rat	Active [60]
	Indomethacin induced gastric ulcer/12.5 mg/kg of crude alkaloid extract - oral	Mouse	Active [60]
Retrorsine 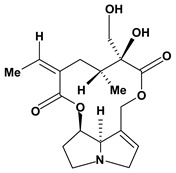	Hypothermic restraint stress induced gastric ulcer/12.5 mg/kg of crude alkaloid extract - oral	Mouse	Active [60]
	Ethanol induced gastric ulcer/12.5 mg/kg of crude alkaloid extract - oral	Rat	Active [60]
	Cysteamine induced duodenal ulcer/12.5 mg/kg of crude alkaloid extract - oral	Rat	Active [60]
	Indomethacin induced gastric ulcer/12.5 mg/kg of crude alkaloid extract – oral	Mouse	Active [60]
Senecionine 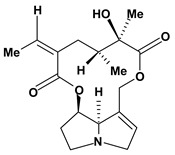	Hypothermic restraint stress induced gastric ulcer/12.5 mg/kg of crude alkaloid extract – oral	Mouse	Active [60]
	Ethanol induced gastric ulcer/12.5 mg/kg of crude alkaloid extract - oral	Rat	Active [60]
	Cysteamine induced duodenal ulcer/12.5 mg/kg of crude alkaloid extract - oral	Rat	Active [60]
	Indomethacin induced gastric ulcer/12.5 mg/kg of crude alkaloid extract – oral	Mouse	Active [60]
Seneciphylline 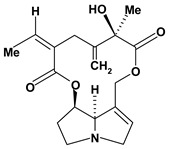	Hypothermic restraint stress induced gastric ulcer/12.5 mg/kg of crude alkaloid extract - oral	Mouse	Active [60]
	Ethanol induced gastric ulcer/12.5 mg/kg of crude alkaloid extract - oral	Rat	Active [60]
	Cysteamine induced duodenal ulcer/12.5 mg/kg of crude alkaloid extract - oral	Rat	Active [60]
	Indomethacin induced gastric ulcer/12.5 mg/kg of crude alkaloid extract - oral	Mouse	Active [60]
Usaramine 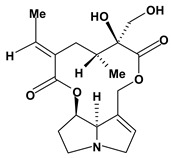	Hypothermic restraint stress induced gastric ulcer/12.5 mg/kg of crude alkaloid extract - oral	Mouse	Active [60]
	Ethanol induced gastric ulcer/12.5 mg/kg of crude alkaloid extract - oral	Rat	Active [60]
	Cysteamine induced duodenal ulcer/12.5 mg/kg of crude alkaloid extract - oral	Rat	Active [60]
	Indomethacin induced gastric ulcer/12.5 mg/kg of crude alkaloid extract - oral	Mouse	Active [60]
**Quinolizidine alkaloids**			
Matrine	Water-immersion stress induced ulcers/25 mg/kg - gastric intubation	Mouse	Active [45]
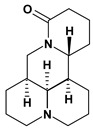	Restraint stress induced ulcers/25 mg/kg - gastric intubation	Mouse	Active [45]
	Water-immersion stress induced ulcers/10 mg/kg - intraperitoneal	Mouse	Weak activity [45]
	Restraint stress induced ulcers/10 mg/kg - intraperitoneal	Mouse	Weak activity [45]
	Water-immersion stress induced ulcers/10 mg/kg - intravenous	Mouse	Active [45]
	Restraint stress induced ulcers/10 mg/kg – intravenous	Mouse	Active [45]
	Stress induced ulcer/50 mg/kg - gastric intubation	Mouse	Active [46]
	Stress induced ulcer/50 mg/kg -Intraperitoneal	Mouse	Active [46]
	Restraint stress induced ulcers/50 mg/kg - gastric intubation	Mouse	Active [47]
	Restraint stress induced ulcers/50 mg/kg - intraperitoneal	Mouse	Weak activity [47]
	Restraint stress induced ulcers/50 mg/kg – intravenous	Mouse	Active [47]
	Pylorus-ligated induced ulcers/50 mg/kg - intraduodenal	Rat	Inactive [45]
	Indomethacin induced ulcers/100 mg/kg - gastric intubation	Mouse	Inactive [45]
	Reserpine induced ulcers/ 50 mg/kg – gastric intubation	Mouse	Active [45]
	Reserpine induced ulcers/25 mg/kg - intravenous	Mouse	Active [45]
Matrine, 13-alpha-hydroxy	Water-immersion stress induced ulcers/* - intragastric	Rat	Active [48]
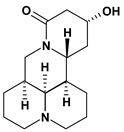	Indomethacin plus alcohol induced gastric ulcers/ * -intragastric	Rat	Active [48]
Matrine, oxy	Pylorus-ligated induced ulcers/*	*	Active [49]
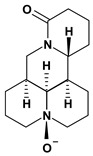	Indomethacin induced ulcers/*	*	Active [49]
	Restraint stress induced ulcers/ * - intraduodenal	Rat	Active [49]
	Water-immersion stress induced ulcers/25mg/kg - gastric intubation	Mouse	Active [45]
	Restraint stress induced ulcers/25 mg/kg - gastric intubation	Mouse	Active [45]
	Water-immersion stress induced ulcers/10 mg/kg - intraperitoneal	Mouse	Active [45]
	Restraint stress induced ulcers/10 mg/kg – intraperitoneal	Mouse	Active [45]
	Water-immersion stress induced ulcers/10 mg/kg - intravenous	Mouse	Weak activity [45]
	Restraint stress induced ulcers/10 mg/kg - intravenous	Mouse	Weak activity [45]
	Stress induced ulcer/50 mg/kg - gastric intubation	Mouse	Active [46]
	Stress induced ulcer/50 mg/kg -intraperitoneal	Mouse	Active [46]
	Restraint stress induced ulcers/50 mg/kg - gastric intubation	Mouse	Active [47]
	Restraint stress induced ulcers/50 mg/kg - intraperitoneal	Mouse	Active [47]
	Restraint stress induced ulcers/50 mg/kg - intravenous	Mouse	Weak activity [47]
	Indomethacin induced ulcers/100 mg/kg - gastric intubation	Mouse	Active [45]
	Reserpine induced ulcers/50 mg/kg - gastric intubation	Mouse	Active [45]
	Reserpine induced ulcers/50 mg/kg - intravenous	Mouse	Inactive [45]
	Pylorus-ligated induced ulcers/* - gastric intubation	Rat	Active [84]
**Steroid alkaloids**			
Pachysamine A, epi	Water-immersion stress induced ulcers/50 mg/kg - intraperitoneal	Mouse	Active [54]
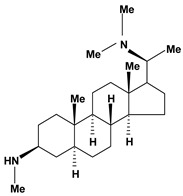	Restraint stress induced ulcers/50 mg/kg – intraperitoneal	Mouse	Active [54]
Pachysandrine A 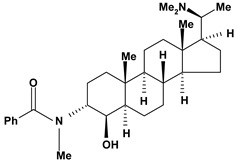	Water-immersion stress induced ulcers/50 mg/kg - intraperitoneal	Mouse	Active [54]
Pachystermine A	Restraint stress induced ulcers/50 mg/kg - intraperitoneal	Mouse	Active [54]
	Water-immersion stress induced ulcers/50 mg/kg - intraperitoneal	Mouse	Active [54]
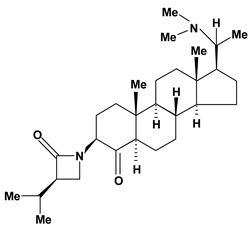			
Spiropachysine	Water-immersion stress induced ulcers/50 mg/kg - intraperitoneal	Mouse	Active [54]
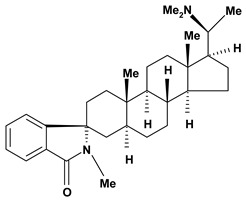	Restraint stress induced ulcers/50 mg/kg - intraperitoneal	Mouse	Active [54]
**Tropane alkaloids**			
Anisodamine	Water-immersion stress induced ulcers/10 mg/kg – intraperitoneal	Rat	Active [41]
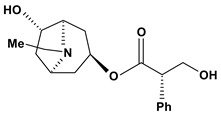	Reserpine induced ulcers/1.5 mg/kg - intraperitoneal	Rat	Active [42]
	Cold stress induced ulcers/2.5 mg/kg - intraperitoneal	Rat	Active [39]
	Indomethacin induced ulcers/0.5 mg/kg - intraperitoneal	Rat	Active [39]
	Acetic acid induced ulcers/10 mg/kg - intraperitoneal	Rat	Active [39]
	Ethanol induced ulcers/25 mg/kg- intragastric	Rat	Active [43]
	Indomethacin induced ulcers/25 mg/kg - intragastric	Rat	Active [43]
	Restraint stress induced ulcers/25 mg/kg - intragastric	Rat	Active [43]
	Pylorus-ligated induced ulcers/25 mg/kg - intragastric	Rat	Active [43]
	Reserpine induced ulcers/1.5 mg/kg - intraperitoneal	Rat	Active [42]
Anisodine	Cold stress induced ulcers/2 mg/kg - intraperitoneal	Rat	Active [39]
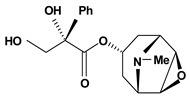	Indomethacin induced ulcers/8 mg/kg - intraperitoneal	Rat	Active [39]
	Acetic acid induced ulcers/32 mg/kg - intraperitoneal	Rat	Active [39]
Atropine	Pylorus-ligated induced ulcers/10 mg/kg - intragastric	Rat	Active [37]
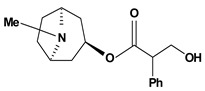	Pylorus-ligated induced ulcers/10 mg/kg - gastric intubation	Rat	Active [38]
	Aspirin induced ulcers/10 mg/kg - gastric intubation	Rat	Active [38]
Cocaine 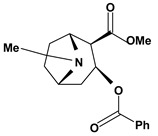	Reserpine induced ulcers/10 mg/kg - oral	Rat	Active [29]
Scopolamine	Cold stress induced ulcers/0.5 mg/kg - intraperitoneal	Rat	Active [39]
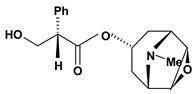	Indomethacin induced ulcers/1 mg/kg - intraperitoneal	Rat	Active [39]
	Acetic acid induced ulcers/8 mg/kg – intraperitoneal	Rat	Active [39]
Scopolamine, methyl 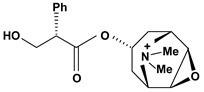	Propionitrile-induced duodenal ulcers/2.5 mg/kg - subcutaneous	Rat	Active [40]
Tropine, n-(4’-ethoxy-carbonyl-phenyl-amine-n’-acetyl)	*/10 mg/kg - intragastric	Rat	Active [83]
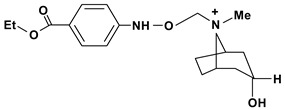			
Tropine, n-(4’-ethoxy-phenyl-amine-n’-acetyl)	*/10 mg/kg - intragastric	Rat	Active [83]
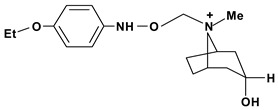			
Tropine, *N*-(4’-sulfamoyl-phenyl-amine-*N*’-acetyl)	*/10 mg/kg - intragastric	Rat	Inactive [83]
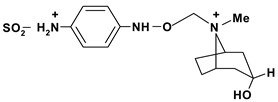			

* = Data not provided

## Conclusions

The studied alkaloids have shown gastroprotetive and antiulcer activities, nevertheless many of them as senecionine, cocaine, nicotine and LSD have several toxic effects on the body such as hepatotoxic, neurotoxic and carcinogenic properties, although some compounds such as morphine (hypnoanalgesic) and ephedrine (nasal decongestant) are more effective in other afflictions. Consequently many alkaloids are not viable for development as gastroprotective drugs or they would need modification of functional groups in their chemical structures for this propose. Nevertheless, they can be used as pharmacological tools in pathophysiological understanding of gastrointestinal diseases, particulalry peptic ulcers, and in the viability of new active compounds for synthesis of new medicines. In this context, it is necessary to support financially the multidisciplinary and interdisciplinary research, mostly those related to natural products enabling the discovery of new antiulcer or gastroprotective pharmaceuticals. 
